# Depressive symptoms, perceived control and quality of life among patients undergoing coronary artery bypass graft: a prospective cohort study

**DOI:** 10.1186/s12912-022-00857-7

**Published:** 2022-04-12

**Authors:** Mohannad Eid AbuRuz, Ghadeer Al-Dweik

**Affiliations:** 1 Department of Nursing, Faculty of Health Sciences, University of Sharjah, Sharjah, United Arab Emirates; 2 Faculty of Nursing, Applied Science Private University, Amman, Jordan

**Keywords:** Coronary artery bypass graft surgery, Depression, Quality of life, Perceived control

## Abstract

**Background:**

Coronary artery bypass graft surgery (CABG) is an intervention directed toward improving the Quality of Life (QoL) for patients with coronary artery disease. Depression can affect QoL negatively among this population. Perceived control (PC) decreased the effect of anxiety on QoL, however, this effect has not been well-studies regarding depression. Therefore, the purpose of this study was to check the effect of depression on QoL among CABG patients and to determine if preoperative PC moderates this effect.

**Methods:**

This was a prospective observational cohort study conducted on a consecutive sample of 200 patients from three hospitals in Amman, Jordan. Depression Anxiety and Stress Scale, Short-Form Health Survey-36, and Arabic version of the Control Attitude Scale-Revised were used to measure depressive symptoms, QoL and PC respectively. Data were analyzed using t test and step wise multiple regression followed by simple slope analysis.

**Results:**

Postoperative Physical Component Summary (PCS) was better than preoperative PCS (mean ± SD: 38.2 ± 9.4 vs. 36.6 ± 9.5, *P* < 0.001). Postoperative Mental Component Summary (MCS) was better than preoperative MCS (mean ± SD: 44.3 ± 11.5 vs. 41.4 ± 11.4, *P* < 0.001). Preoperative depression was higher than postoperative depression; (mean ± SD: 12.8 ± 6.8 vs.11.1 ± 6.7, *P* < 0.01). Simple slope analysis was significant (simple slope = 0.41, *t* = 6.1, *P* < 0.001), indicating the moderating effect of PC.

**Conclusion:**

Patients undergoing CABG surgery had poor QoL and high levels of depression. Perceived control moderated this relationship and improve QoL. Assessing depression levels and implantation of interventions to enhance perceived control levels prior to operation might improve QoL.

## Background

Coronary artery bypass graft surgery (CABG) is a well-known operation for the treatment of coronary artery disease in both developed and developing countries [1, 2]. Due to the technological development and advancement in the (pre and post) operative care, mortality rates after CABG have declined over decades with 98% survival rate during the first 4 months after surgery [2–4]. However, mortality rates alone do not reflect the appropriate outcome measures post CABG [2, 3, 5]. Thus, the goals of coronary artery disease treatment should be more dedicated not only on prolonging life, but also on relieving symptoms, improving physical and mental functional status, and performing daily activities [2]. Most of these outcomes are measured subjectively through Quality of Life (QoL) [2, 6]. For this reason, researchers start to have much interest regarding QoL after CABG [3, 7–10].

It has been shown that CABG was associated with benefits that include symptom relief, improvement in QoL, reduce disability, and extended survival [1, 4, 11–14]. Contrary, other studies have shown that CABG might lead to undesirable consequences of lengthy or poor recovery, problems of physical activity, sleep disturbances, increase morbidity ending with poor QoL post operatively [3, 11, 15, 16]. Estimates indicated that a percentage between 15.7 to more than 25% of post CABG patients reported that their QoL decreased compared to preoperative period [4, 17–20]. It is worthy to note that also some studies reported that QoL did not changed after the operation [17, 21]. Therefore, it is necessary to identify factors that might affect outcomes, namely QoL, after surgery accordingly appropriate interventions can be implemented.

Quality of Life is a multi-dimensional aspect of the human’s perception of the physical, psychological and social aspects of life which might be affected by a disease process and its treatment [3, 8]. Also, QoL may be changed by a person’s experiences, attitudes, anticipations, and perceptions. In addition, specifically, post CABG, QoL might be affected by: age, gender, length of stay (LoS), depression, perceived control (PC), and many others.

There are inconsistent results regarding the effect of gender on QoL post CABG. Some investigators reported that female gender was a predictor for poor QoL, while others reported the opposite [7, 22]. Male gender was an improvement predictor to QoL in other studies [5, 23, 24]. Other investigators found that there was no significant difference in QoL based on gender [25]. Longer LoS was associated with lower levels of functional capacity which in turn decreased QoL [26]. In a systemic review checking if CABG could improve QoL in elderly patients; it was found that ICU LoS greater than 2 days was associated with lower levels of QoL [8].

The outcomes and recovery after acute cardiac events/ procedures (i.e. CABG and acute myocardial infarction) depend on diverse physiological and psychological factors. The role that psychological factors play in the recovery was as important as or even more important than physiological factors [11, 27–29]. Depression is one of the most prevalent psychological factors in the pre-operative period that affecting QoL post-operatively. Among CABG patients, the reported rates of pre-operative depression ranged from 14 to 60% [11, 13, 14, 30–33]. Despite that, health care providers failed in screening more than 50% of their patients undergoing CABG for depressive symptoms [11, 34, 35].

Different studies have shown that preoperative depression increased anginal pain, occurrence of delirium, and prolonged postoperative LoS [4, 11–14, 34, 36]. Studies checking the effect of pre-operative depression on QoL early after CABG are limited [37]. Per-operative depression was correlated with poor QoL post CABG including bodily pain, vitality, social functioning, emotional role function and general health [37]. Other studies showed that per-operative depression was associated with poor QoL on the long term up to 5 years after the operation [4, 16, 19, 20, 38, 39]. Therefore, identifying factors that has a moderating effect on the relationship between depression and QoL is important.

Perceived control has been defined as “an individual’s belief that he or she has the resources required to cope with negative events in a way that positively influences their adversive nature” [40]. It has been shown that PC has positive effects among diverse cardiac populations including post CABG, acute myocardial infarction, cardiac transplant and heart failure [1, 28].

Higher levels of PC were associated with better QoL and lower levels of depressive symptoms among 149 CABG patients 6–8 weeks following the operation after controlling for all covariates [4]. Moreover, high levels of PC in the per-operative period, were associated with lower levels of anxiety and depression post operatively among 155 CABG patients [41]. In addition, PC moderated the relationship between anxiety and LoS among 250 post CABG patients [1]. Together, these results indicate that PC plays a key feature in the immediate recovery for those patients, affecting their QoL positively. On the other hand, lower levels of PC were associated with poor QoL and higher levels of depressive symptoms [42]. Furthermore, to our knowledge, only one study reported that PC did not predict depressive symptoms nor QoL after cardiac surgery. However, this study included small sample size of only 56 patients [43]. Therefore, the major purpose of this study was to check the effect of depression on QoL among CABG patients and to determine if preoperative PC moderates this effect.

### Research hypotheses

(H1) preoperative depression is higher than postoperative depression; (H2) postoperative QoL is better than preoperative QoL; (H3) prior and after; surgery female patients have higher levels of depression and lower levels of QoL compared to male patients; (H4) preoperative depression and PC predict postoperative QoL after controlling for sociodemographic and clinical variables; (H5) preoperative PC moderates the relationship between depression and QoL.

## Methods

### Design, sample, and setting

This was a prospective observational cohort study conducted at (one governmental, one teaching, and one private) hospitals in Amman, Jordan. A consecutive sample of all patients who met the following inclusion criteria were included: (1) adult patients older than 18 years, (2) elective CABG operation, (3) free from depression diagnosis (as confirmed by a psychiatrist), (4) not on anti-depressant medications, (5) able to read and write in Arabic. Patients with other chronic diseases including rheumatoid arthritis, multiple sclerosis, and Parkinson were excluded. Furthermore, all open heart surgeries other than CABG were excluded.

**Fig. 1:**
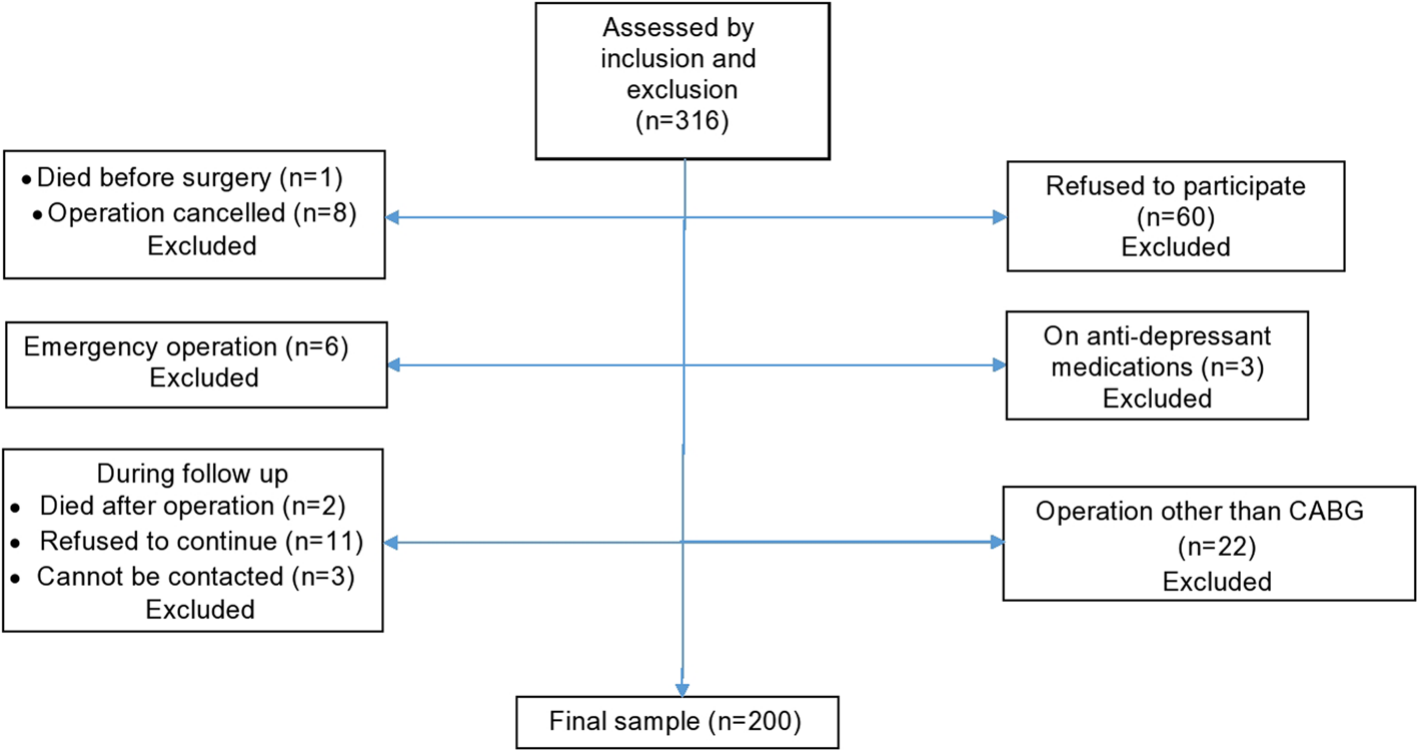
Patients flow diagram

Power analysis was used to make sure that sample size was enough to run the appropriate statistical tests which were: paired t test for H1 and H2, independent t test for H3, and multiple regression for hypotheses 4 and 5, with 13 independent variables. Other assumptions were alpha coefficient of 0.05, power of 0.95, and a medium effect size between depression and QoL. Based on that, the needed sample size was 54 patients for H1 and H2, 198 patients for H3, and 184 patients for H4 and 5. Recruitment continued until the sample reached 200 patients (Fig. 1). No significant differences were found between those who responded and those who were excluded in terms of sociodemographic and clinical characteristics.

### Procedure

At the cardiology clinics of the selected hospitals, trained cardiovascular research assistants holing a master’s degree in critical care approached every patient for planed open heart surgery and screened them using the inclusion and exclusion criteria. If the patient met the criteria, research assistants explained the study in detailed and let the patients sign an informed consent form including a permission to review their medical records. At this meeting (within 1 week prior to operation), the patients answered sociodemographic questionnaire about age, gender, (marital, smoking and working) status, the Depression Anxiety and Stress Scale (DASS-21), Control Attitude Scale-Revised (CAS-R), and Short-Form Health Survey-36 (SF36). Post discharge, research assistants reviewed medical records to measure hospital length of stay, history of comorbidities (i.e. Diabetes Mellitus (DM), Hypertension (HTN), previous angina previous myocardial infarction), Left Ventricular Ejection Fraction (LVEF), and body mass index (BMI; kg/m^2^). Three months later, research assistants called the patients by phone and filled with them the DASS and the SF 36 as a follow up measure.

### Measurement of variables

*Depression:* was measured using the Arabic version of DASS-21 which is a 21-item self-report instrument. The original instrument was developed by Lovibond and Lovibond (1995) [44]. The instrument has three subscales each one consists of 7 items with 4 Likert scale options measuring depression, anxiety and stress symptoms over the last week. The scores for each subscale ranges from 0 to 21 with higher scores indicating higher symptoms frequency and severity. For this study, only the depression subscale was used. The cutoff points for depression subscale are 0–4 normal, 5–6 mild, 7–10 moderate, ≥ 11 sever.

The original instrument was found to be valid and reliable with Cronbach’s alpha for depression subscale was 0.91 [45]. The Arabic version psychometric proprieties were supported at different studies with different populations. In Jordan, where the current study was performed, two studies supported the psychometric proprieties of the DASS, with depression’s subscale Cronbach’s alpha ranged from 0.77–0.88 in the first study [46] and 0.90 in the second [47]. Among 220 immigrant participants in Sydney, Australia, the study supported the validity and reliability of the DASS using factor analysis, the universality of depression across cultures, and the ability to use the English norms for Arab populations [48]. In this study Cronbach’s alpha was 0.91.

*Perceived Control:* was measured by the Arabic version of the CAS-R. This instrument has been used in previous studies among CABG patients. This version demonstrated sound psychometric properties with Cronbach’s α of 0.75 [1, 28]. In this study the Cronbach’s alpha was 0.89. This instrument is composed of eight Likert scale items with five-option answers from 1 “totally disagree” to 5 “totally agree”. The possible range for the total score is from 8 to 40. Higher scores reflect greater levels of PC [1, 28]. Median has been used by researchers to classify participants with high and low PC since there are no published mean norms [1, 28].

*Quality of Life:* was measured by the Arabic version of the SF36 which a generic measure of 8 domains of health namely: physical functioning, role physical functioning, role emotional functioning, mental health, vitality, role social functioning, bodily pain, and general health. The raw scores of each domain is transformed to 0–100 scale with higher scores indicating better QoL in each domain. If patients scored below 47, then they were considered to have poor QoL [49–51].

For the purposes of this study we used the two major summaries of the QoL measured from these domains: The Physical component summary (PCS) including (physical functioning, role physical functioning, body pain, and general health), and the Mental Component Summary (MCS), representing; role emotional functioning, vitality, mental health, and social functioning [49–51]. The mimimum Cronbach’s α for the Arabic version was 0.71 and the highest was 0.94 [49–52].

*Ethical consideration:* The study was approved by the institutional review board committee at Applied Science Private University, Amman, Jordan and from all institutions before data collection. Informed consent was signed by all participants who agreed to participate including a permission to review their medical records. Patients were ensured that their participation is totally voluntary, and they can withdraw at any time. Confidentiality was maintained by assigning identification numbers to participants, keeping data in a locked cabinet with access only by the principle investigator, and the use of aggregate data for publication.

### Data analyses

SPSS version 25 was used for the analysis. H1 (preoperative depression is higher than postoperative depression) and H2 (postoperative QoL is better than preoperative QoL) were tested by paired t test. H3 (prior and after surgery; female patients have higher levels of depression and lower levels of QoL compared to male patients) was tested by independent sample t test. H 4 (preoperative depression and PC predict postoperative QoL after controlling for sociodemographic and clinical variables) and H5 (preoperative PC moderates the relationship between depression and QoL) were tested by stepwise multiple regression, followed by simple slope analysis of ±1 SD from the mean. In the first model, PC scores, depression scores, and all sociodemographic and clinical variables were entered. In the second model, the interaction between centralized PC and depression scores was included.

## Results

### Clinical and sociodemographic characteristics

Among the 200 participants, 70 (35%) were females, and 155 (77.5%) were married. Most of the sample had angina and nearly half of them had previous AMI. On the other hand, more than half of the sample were currently smokers. Other characteristics are presented in Table 1.

**Table 1:** Clinical and sociodemographic characteristics (*N* = 200)

Characteristics	Mean ± SD or n (%)
Age	63.7 **±** 8.4
Gender	
Male	130 (65.0)
Female	70(35.0)
Marital status	
Married	155 (77.5)
Single/divorced/widowed	45 (22.5)
Currently working	80 (40.0)
History of HTN	166 (83.0)
History of DM	121 (60.5)
History of previous AMI	103 (51.5)
History of previous angina	187 (93.5)
Smoking History	
Never smoked	50 (25.0)
Current smoker	110 (55.0)
Former smoker	40 (20.0)
Post-operative hospital LOS	11.2 ± 10.4
BMI (kg/m^2^)	25.8 ± 4.4
LVEF	47.5 ± 8.9
Total CAS-R	21.3 ± 4.3
Pre-operative depression	12.8 ± 6.8
Normal	35 (17.5)
High	165 (82.5)

*Abbreviations*: *AMI* Acute myocardial infarction, *BMI* Body mass index, *CAS-R* Control attitude scale revised, *DM* Diabetes mellitus, *HTN* Hypertension, *SD* standard deviation

### Hypotheses testing

H1 (preoperative depression is higher than postoperative depression) and H2 (postoperative QoL is better than preoperative QoL) were tested by paired t test. The results showed that preoperative depression was higher than postoperative depression; (mean ± SD: 12.8 ± 6.8 vs.11.1 ± 6.7, *P* < 0.01). Postoperative PCS was better than preoperative PCS (mean ± SD: 38.2 ± 9.4 vs. 36.6 ± 9.5, *P* < 0.001). Postoperative MCS was better than preoperative MCS (mean ± SD: 44.3 ± 11.5 vs. 41.4 ± 11.4, *P* < 0.001). H3 (prior and after surgery; female patients have higher levels of depression and lower levels of QoL compared to male patients) was tested by independent sample t test. The results are shown in Table 2. It is worthy to note that there were no differences between female and male patients in any of sociodemographic and clinical chrastatistics expect for levels of LVEF and length of stay. Compared to male patients; female patients have lower levels of LVEF and longer length of stay; (mean ± SD: 46.1 ± 9.1 vs. 48.6 ± 8.5, *P* < 0.05), and (mean ± SD: 12.7 ± 12.3 vs. 10.5 ± 9.7, P < 0.05) respectively. H 4 (preoperative depression and PC predict postoperative QoL after controlling for sociodemographic and clinical variables) and H5 (preoperative PC moderates the relationship between depression and QoL) were tested using multiple regression followed by simple slope analysis. The results of the regression analysis are presented in Tables 3 and 4.

**Table 2:** (Pre and post) operative comparison of depression and quality of life between men and women using independent sample t test (*N* = 200)

	Preoperative			Postoperative		
	Mean ± SD		*P* value	Mean ± SD		*P* value
	Male	Female		Male	Female	
**Depression**	11.3 ± 6.6	14.6 ± 7.6	< 0.001	9.4 ± 6.4	13.1 ± 6.9	< 0.001
**PCS**	39.0 ± 8.9	33.6 ± 9.3	< 0.001	41.1 ± 8.9	34.3 ± 8.7	< 0.001
**MCS**	44.0 ± 10.4	38.0 ± 11.6	< 0.001	46.5 ± 10.4	40.6 ± 11.7	< 0.001

*Abbreviations*: *PCS* physical component summary, *MCS* mental component summary, *SD* standard deviation

**Table 3:** Predictors of postoperative quality of life/PCS by stepwise regression analysis (*N* = 200)

Variable	Model 1		Model 2	
	Standardized β	t	Standardized β	t
Female gender	−.18*	2.6	−.17**	−2.5
Pre-op depression	−.31**	5.3	−.35**	− 6.5
PC scores	.26**	4.9	.25**	4.31
Depression scores ×PC			.37**	6.6
Adjusted R^2^	0.28		0.38	
F	8.0		11.61	

*Abbreviations*: *BMI* body mass index, *DM* diabetes mellitus, *LVEF* Left Ventricular Ejection Fraction; myocardial infarction, *PC* perceived control, *PCS* physical component summary

∗*p* < 0.05; ∗∗*p* < 0.01. In the first step, gender, age, working status, marital status, history of hypertension, DM, previous MI, previous angina, BMI, PC scores, LVEF, and preoperative depression scores were entered as independent variables. In the second step, the interaction between centerized PC and preoperative depression was included

**Table 4:** Predictors of postoperative quality of life/MCS by stepwise regression analysis (*N* = 200)

Variable	Model 1		Model 2	
	Standardized β	t	Standardized β	t
Female gender	−.26**	−2.9	−.31**	−4.0
Pre-op depression	−.25**	−2.8	−.22**	−2.4
PC scores	.16*	1.9	.17*	2.2
Depression scores ×PC			.35**	4.3
Adjusted R^2^	0.26		0.39	
F	7.0		13.8	

*Abbreviations*: *BMI* body mass index, *DM* diabetes mellitus, *LVEF* Left Ventricular Ejection Fraction; myocardial infarction, *PC* perceived control, *MCS* mental component summary

*p < 0.05; **p < 0.01. In the first step, gender, age, working status, marital status, history of hypertension, DM, previous MI, previous angina, BMI, PC scores, LVEF, and preoperative depression scores were entered as independent variables. In the second step, the interaction between centerized PC and preoperative depression was included

Being female reduced PCS by 0.17 unit. Everyone unit increase in preoperative depression level reduced PCS by 0.35 unit. PC has a positive effect; everyone unit increase in PC increased PCS by 0.25 units. The interaction term in Model 2 was significant. Furthermore, the R^2^ change between Model 1 and Model 2 was significant (ΔR^2^ = 0.10, *P* < 0.01). These results indicated that PC was a significant moderator in the relationship between preoperative depression and QoL/PCS.

Being female reduced MCS by 0.31 unit. Everyone unit increase in preoperative depression level reduced MCS by 0.22 unit. PC has a positive effect; everyone unit increase in PC increased MCS by 0.17 units. The interaction term in Model 2 was significant. Furthermore, the R^2^ change between Model 1 and Model 2 was significant (ΔR^2^ = 0.13, *P* < 0.01). These results indicated that PC was a significant moderator in the relationship between preoperative depression and QoL/MCS.

In simple slope analysis; when the effect of the independent variable (preoperative depression) on the dependent variable (QoL/PCS) when the moderator (PC) is high (1 SD) above and low (1 SD) below the mean was done, preoperative depression decreased QoL/PCS when the moderator (PC) was low (simple slope = 0.41, *t* = 6.1, *P* < 0.001), and this turns to be insignificant when the moderator (PC) was high. These results indicated that PC worked as a moderator between preoperative depression and QoL/PCS. The same results we found when that was checked in relation to the MCS, (simple slope = 0.42, *t* = 6.9, *P* < 0.001) which also indicated that PC worked as a moderator between preoperative depression and QoL/MCS.

## Discussion

This is the first study that was designed specifically to check if PC moderated the effect of depression on QoL after CABG in a developing country. Also, it aimed to check if preoperative depression was higher than postoperative one, and whether it predicted QoL after the operation. Similarly, the study checked if postoperative QoL was higher than preoperative QoL. Additionally, the study aimed to determine if there were differences in QoL and depression based on gender. The results indicated that: (1) PC moderated the effect of depression on QoL, (2) preoperative depression was higher than postoperative depression and higher levels of depression worsen the QoL after operation, (3) postoperative QoL was better than preoperative QoL, and (4) females had higher levels of depression and therefore had lower levels of QoL compared to males.

### Depression and QoL post CABG

The results showed that higher levels of depression were associated with lower levels of QoL. This result is consistent with previous studies [16, 20, 37–39]. Even in one study depression has been shown to have a powerful effect compared to ejection fraction and ischemia [53]. Similarly, depression has been shown to have negative effect on QoL even when the operation itself was successful [54]. Possible explanations why depression might lead to lower levels of QoL include but not limited to: (1) physiologically, depression increased the incidence of inflammation due to an increase in the secretion of pro-inflammatory cytokines by stimulating hypothalamic pituitary adrenal access [11, 13, 14, 55]. Moreover, high levels of depression were associated with higher incidence of delirium [37, 56–58], (2) Socially or interactively, patients with high levels of depression showed behavioral alterations as poor hygiene, unhealthy nutritional habits including drinking large amount of alcohol and lack of medication adherence [13, 37, 55, 59].

The results of this study also showed that the preoperative depression levels were high and higher than those in the postoperative period. This result is consistent with previous studies [11, 13, 14, 55]. Usually, patients who are planned for CABG surgery will be physically tried and complaining form diverse symptoms that affect their abilities to perform activities of daily living as angina, shortness of breath and fatigue. Generally, CABG operation will result in resolving these symptoms gradually, and therefore postoperative depression levels were lower. In addition to that, the successful performance of the operation itself will enhance the reduction of depression among those patients.

### Quality of life before and after CABG

Consistent with previous studies, this study indicated that postoperative QoL was better than preoperative QoL for both men and women [5, 8, 22, 24, 60, 61], and this is affecting MCS more than PCS [25]. Despite that, it is worthy to note that both QoL were lower than the cutoff point of 47 indicating that the QoL for those patients still poor. Possible explanations why there was improvement in the QoL might be: (1) reduction of the level of depression at the postoperative period, and since higher levels of depression were associated with lower levels of QoL it is expected that postoperative QoL will be higher than preoperative one, (2) previous studies indicated that CABG operation will result in resolving of major signs and symptoms of coronary artery disease, like angina, shortness of breath and fatigue [1, 4, 11–14]. Resolution of these symptoms help patients participating more in activities of daily living, socialization and returning to work earlier. These in turn might help in improving QoL.

Other studies, however, showed that there was a reduction in QoL in the postoperative period [4, 62] or there was no change at all in the QoL [21]. For instance, Kidd at al [4] showed that there was a reduction in the PCS in the postoperative period compared to the preoperative one. A possible explanation for this difference might be the timing when data collection was performed in these studies. In current study, data were collected 3 months after the operation, while in Kidd et al. study this was 6-8 weeks after the operation. Patients post CABG, in the early phase (up to 2 months), usually complain from problems of physical activity, socialization, and sleep disturbances [3, 15]. In a literature comparing the QoL after CABG versus PCI, it was found that the QoL for patients 1 month after the procedure was better for PCI patients compared to CABG. However, this was the opposite for 6 months period. Moreover, as depression decrease overtime after surgery, QoL usually improved since high levels of depression were associated with lower QoL.

In a literature review [60] including 45 studies about postoperative QoL of older people following cardiac surgery, among which 9 only were prospective studies, the majority of the results indicated that there was an improvement in the QoL postoperatively. However, 8–19% of these studies showed that there was a decline in the QoL postoperatively. This difference might be due to the enrollment of the old-aged people and the nature of the design which is mostly retrospective compared to the current prospective one.

### Gender, depression and QoL

The results of this study showed that female patients have higher levels of depression and lower levels of QoL compared to male patients. Diverse studies among cardiac populations including CABG [11, 34], acute myocardial infarction and heart failure showed that females had higher levels of depression compared to males [11, 27, 29, 34, 63–65]. Reasons behind that might be: (1) the inverse association between depression and left ventricular ejection fraction that has been demonstrated among coronary artery disease [65, 66], heart failure [65, 67], and acute myocardial infarction [65, 68]. In this study, males had significantly higher levels of left ventricular ejection fraction compared to females; (2) among cardiac populations, females have higher levels of fatigue compared to males, which has a positive correlation with depression; (3) new research area is focusing on the association between fetal exposure and depression; and (4) higher perioperative depression levels among females compared to males; which might explain the higher levels at the postoperative period.

Regarding QoL, females also have lower levels compared to males which might be due to higher levels of depression and lower levels of left ventricular ejection fraction. Given that females had higher levels of depression, and higher depression levels were associated with lower levels of QoL, it is suspected that they will have lower levels of QoL. Previous studies demonstrated that low levels of left ventricular ejection fraction were a strong predictor for poor QoL [49–51]. Again, since females had lower levels of left ventricular ejection fraction, it is unsurprising that they have lower levels of QoL compared to males.

### PC, depression, and QoL (the moderating effect)

Like previous studies checking the moderating effect of PC among CABG patients, and among depression [41, 55], this study showed that PC has a positive moderating effect on the relationship between depression and QoL. The association between PC and QoL is not well studied in the literature. However, studies have shown that low levels of PC were independent predictor of higher depressive symptoms [4]. Additionally, low levels of PC were associated with elevated stress and helplessness leading to negative feelings and communicative outcomes [4]. There is also considerable testimony that paucity of control has undesirable consequences on biological activities related to health, including cardiovascular activity, neuroendocrine responses, and immune processes [4]. Further research is warranted to explain how these processes function and affecting QoL in CABG patients.

Studies checking the moderating effect of PC on CABG patients are limited [1, 55]. The first study showed that PC moderated the relationship between depression and length of stay post CABG [55]. The second study showed the same results reading the relationship between anxiety and length of stay post CABG [1]. It is worthy to note that previous studies demonstrated a relationship between negative emotion and longer length of stay at the hospital. Reduction of hospital length of stay might have better outcomes on patients QoL explaining how PC worked in improving the QoL for those patients.

Among other cardiac populations, it has been shown that PC had a moderating effect on the relationship between depression and complications after acute myocardial infarction [69] and between anxiety and complications after acute myocardial infarction [28]. In Heart failure patients, PC controlled depressive symptoms and result in better QoL [70, 71].

## Conclusion and implication to practice

Patients undergoing CABG surgery had poor QoL and high levels of depression. Being female and having high levels of depression negatively affecting the QoL for this population. PC moderated this relationship and improve QoL. Assessing depression levels and implantation of interventions to enhance PC levels prior to operation especially among females might improve QoL.

### Limitations

Some of the information for this study was collected from medical records which depends on documentation from 3rd personnel. The duration of the follow up is only 3 months. Longer follow up duration is recommended. Even it is out of the scope of this study, we did not scrutinize the specific underlying mechanism of how PC moderated the effect of depression on QoL for this population. Further studies covering this purpose is recommended.

## Data Availability

The datasets analyzed in the current study available from corresponding author on reasonable request.

## Abbreviations

BMI: Body Mass index
CABG: Coronary artery bypass graft surgery
CAS-R: Control Attitude Scale-Revised
DASS: Depression Anxiety and Stress Scale
DM: Diabetes Mellitus
HTN: Hypertension
LVEF: Left Ventricular Ejection Fraction
LoS: Length of Stay
MCS: Mental Component Summary
PC: Perceived Control
PCS: Physical Component Summary
SF36: Short-Form Health Survey-36
QoL: Quality of Life
